# Canary in the coliform mine: Exploring the industrial application limits of a microbial respiration alarm system

**DOI:** 10.1371/journal.pone.0247910

**Published:** 2021-03-04

**Authors:** Wendy Stone, Tobi M. Louw, Marthinus J. Booysen, Gideon M. Wolfaardt

**Affiliations:** 1 Water Institute and Department of Microbiology, Stellenbosch University, Stellenbosch, South Africa; 2 Department of Process Engineering, Stellenbosch University, Stellenbosch, South Africa; 3 Department of E&E Engineering, Stellenbosch University, Stellenbosch, South Africa; 4 Department of Chemistry and Biology, Ryerson University, Toronto, Canada; Chinese Academy of Sciences, CHINA

## Abstract

Fundamental ecological principles of ecosystem-level respiration are extensively applied in greenhouse gas and elemental cycle studies. A laboratory system termed CEMS (Carbon Dioxide Evolution Measurement System), developed to explore microbial biofilm growth and metabolic responses, was evaluated as an early-warning system for microbial disturbances in industrial settings: in (a) potable water system contamination, and (b) bioreactor inhibition. Respiration was detected as CO_2_ production, rather than O_2_ consumption, including aerobic and anaerobic metabolism. Design, thresholds, and benefits of the remote CO_2_ monitoring technology were described. Headspace CO_2_ correlated with contamination levels, as well as chemical (R^2^ > 0.83–0.96) and microbiological water quality indicators (R^2^ > 0.78–0.88). Detection thresholds were limiting factors in monitoring drinking water to national and international standards (0 CFU/100 mL fecal coliforms) in both open- (>1500 CFU/mL) and closed-loop CO_2_ measuring regimes (>100 CFU/100 mL). However, closed-loop detection thresholds allow for the detection of significant contamination events, and monitoring less stringent systems such as irrigation water (<100 CFU/mL). Whole-system respiration was effectively harnessed as an early-warning system in bioreactor performance monitoring. Models were used to deconvolute biological CO_2_ fluctuations from chemical CO_2_ dynamics, to optimize this real-time, sustainable, low-waste technology, facilitating timeous responses to biological disturbances in bioreactors.

## Introduction

Carbon dioxide production is a universal biological indicator of respiration, and is a parameter indicative of life, often harnessed as an indicator of ecosystem health [1, 2]. Pursuing a similar bird’s-eye-view, Kroukamp and Wolfaardt [3] developed the Carbon Dioxide Evolution Measurement System (CEMS), which harnesses microbial CO_2_ production to study whole-biofilm metabolic profiles. Unlike standard respirometry [4], CO_2_ rather than O_2_ is monitored as the by-product of glycolysis and the Krebs cycle. This metabolic pathway is common to aerobic and anaerobic metabolism, and the measurement of CO_2_ fluctuation is also indicative of photosynthetic metabolism. The system traps biofilm-produced CO_2_, passing it over an analyzer on CO_2_-free sweeper gas, and logging the data. Online platforms allow for remote, real-time monitoring of disturbances. It is used primarily to study pure culture laboratory biofilms, and has generated information on the relationship between biofilm inoculum, nutrients and metabolism [5, 6]; metabolic responses to antibiotic treatments [7, 8] and to track cellulolytic activity [9]. This design has also been adapted to a closed-loop CO_2_ accumulation system to monitor low-level microbial metabolic patterns during desiccation [10].

Understanding microbial activity at community and species levels does require the genetic, proteomic and metabolic profiling of the specific active and dormant organisms within an ecosystem. However, the traditional narrow focus on pathogenic species in water quality monitoring may well contribute to the many failures in detecting outbreaks, as indigenous communities could mask pathogens in our efforts to detect them. There are practical benefits and unique perspectives facilitated by the immediate and high-level availability of whole-ecosystem metabolic footprints. This is particularly true when coupled with modelling, which can predict many of the physico-chemical CO_2_ fluctuations, resolving the biological data from total CO_2_ information.

We evaluate the potential of using CEMS as an alarm in commercial and industrial settings, analogous to the miner’s canary. The technology is assessed as an indicator in two similar but converse undesirable industrial scenarios: (1) the contamination of potable water systems, and (2) the inhibition of bioreactors for the treatment of waste- or industrial service water. The second scenario is especially relevant, since failure of wastewater treatment systems can result in serious environmental pollution, due to unexpected inflow of chemicals that inhibit growth, or careless incorporation of industrial waste streams without taking biodegradability into account. For instance, wastewater loading impact is commonly calculated based on COD rather than contaminant toxicity.

Any fluctuations of CO_2_ production above a pre-determined minimum respiration rate can act as a high-level indicator of microbial contamination events in potable water systems, providing maintenance staff a warning to investigate more fully. Whether water is stored in a tank or distributed in pipes in systems such as dental chairs, microbial contamination of potable water is often the cause of disease outbreaks [11, 12]. With growing water scarcity challenges in drought-ridden areas, and the associated popularity of decentralized water systems gaining traction particularly in developing countries, remote monitoring for microbial contamination is paramount. Moreover, the microbiological monitoring of drinking water is limited by time (culturing), cost and expertise (quantitative real-time PCR, enzyme assays), which hampers the public provision of decentralized point-of-use water treatment systems. Most of these techniques involve the mass use of disposable reagents and equipment, a critical consideration in the current environmental waste crisis. CEMS has the benefit of real-time and remote monitoring of microbial activity, with equipment that is long-lasting and energetically sustainable using solar power, generally liberally available in drought-ridden areas.

In the converse scenario, in the biological treatment of sewage and industrial wastewater, microbial activity is responsible for converting dissolved pollutants into the gas phase, solid phase for removal as biomass, or into more innocuous chemical forms. Their metabolic activity can simultaneously act as a reliable indicator of changes in the waste treatment process, including changes in chemical composition (nutrient spikes, toxins, pH), temperature and failure of mixing (oxygenation vs anaerobiosis). Optimal functionality should be associated with a quantifiable CO_2_ steady-state, and predictable fluctuation thresholds. Companies that utilize waste treatment bioreactors can be fined extensively if the reactors fail and toxic waste is released into water sources, recorded in governmental non-compliance records [13–15]. These fines are proportional to waste volume, and an immediate, durable indicator of bioreactor failure due to the disruption of microbial metabolism is both environmentally and financially valuable.

In this study, the thresholds of this CO_2_ monitoring technology were shown to limit use as an early-warning system for potable water, but were sufficient to meet agricultural water standards. The system was an effective alarm for bioreactor disturbances. Modelling was employed to tease out physico-chemical CO_2_ fluctuations from the metabolic response of the microbial aggregate, which is the active role player in waste remediation.

## Materials and methods

### Sampling: River water and wastewater

The CEMS performance was evaluated in two different contexts: for the monitoring of clean water, which should ideally contain very low levels of microbial contamination, and the monitoring of an active bioreactor. To assess the former, river water samples were collected from the polluted Plankenbrug River, Stellenbosch, South Africa (-33.933983; 18.85095) [16]. This polluted river water was diluted with pure water to mimic a range of pollution levels, from low to high. For the latter, Return Activated Sludge (RAS) samples were collected from a municipal wastewater treatment works in Cape Town, South Africa.

### Canary CEMS for potable water storage systems

**Fig 1:**
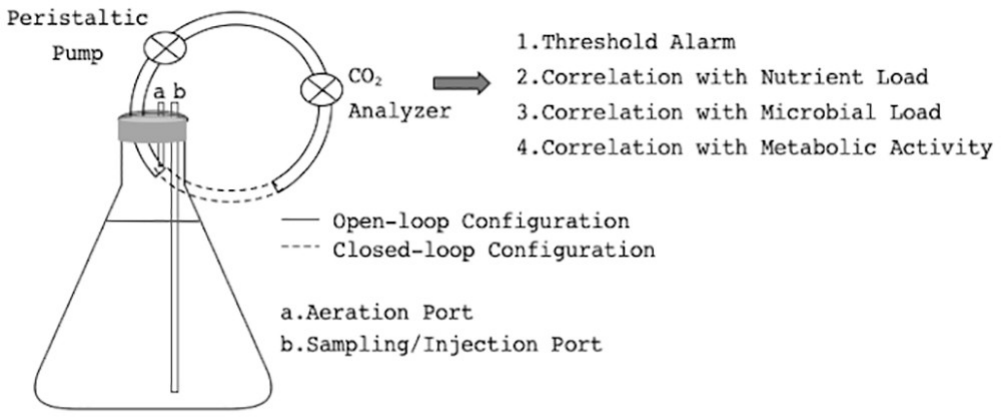
Schematic of the laboratory-scale canary CEMS alarm system. To assess minimum thresholds for whole-reactor CO2 monitoring, as an indicator of (1) water storage contamination events and (2) metabolic disturbances of bioreactors.

Sterile 5 L Erlenmeyer flasks were filled with river water, mixed continuously to ensure aeration, and no additional nutrients added. The flasks were sealed and the CEMS tube for headspace gaseous analysis was placed directly above the liquid surface. A small air inlet port prevented negative pressure. The headspace gas was transferred via a peristaltic pump (flow rate = 15 mL/min; carrier gas = ambient air) through a LiCor CO_2_ analyzer (Campbell Scientific, Stellenbosch, South Africa), with continuous data logging per second (Fig 1 –open loop). Thus, CO_2_ measurements were investigated as a difference between atmospheric and reactor CO_2_ levels, rather than absolute CO_2_ production, as measured in CO_2_-free air in previously described CEMS studies [5]. Atmospheric baselines were measured repeatedly on different days (10 days, minimum of 5 hrs per baseline), to assess variation. Two sensors were used throughout the experiment, calibrated every 3–7 days with a CO_2_ gas standard (AFROX, Cape Town, South Africa), and randomly exchanged between control and experimental reactors throughout the study. The random sensor exchange was to monitor potential sensor drift and for normalizing, preventing the constant exposure of each sensor to either high or low CO_2_ concentrations. Averages and standard deviations were calculated in Microsoft Excel for Mac (v 15.25), throughout the study.

#### Headspace effect

The effect of headspace volume on CEMS sensitivity was evaluated at different volumes: (1) 0.9 L headspace (5.0 L liquid), (2) 0.55 L headspace (5.35 L liquid) and (3) 0.25 L headspace (5.65 L liquid), as described in Table A1 in the S1 Appendix. In order to assess lower detection thresholds, both undiluted and 25% samples (v river water/v sterile tap water) were evaluated. The average total microbial CO_2_ production [ppm CO_2_ –ppm CO_2_ (ambient control)] was plotted against headspace volume. Liquid surface area was recorded per treatment. The control was an identical reactor containing sterile (autoclaved, 121 °C, 15 psi, 30 min) river water. All further measurements were assessed at 0.9 L headspace (5.0 L liquid). The differences between means were compared individually at decreasing headspaces, between (1) & (2), and (2) & (3), for diluted and undiluted reactors.

#### Minimum detection threshold

In order to correlate CO_2_ accumulation and SANS241 parameters [17], headspace accumulation of CO_2_ in the river water reactors was measured (0.9 L headspace), along with the chemical and microbiological profiles (described below). To determine minimum thresholds, river water samples were subsequently diluted with sterile tap water (0%, 1%, 10%, 25%, 50%, 100% (v river water/v sterile tap water)), representing an increasingly contaminated potable water system, and evaluated for the same metabolic, chemical and microbiological profiles. Measurements were done for separate, triplicate experiments. These were assessed for linear correlations in Microsoft Excel for Mac (v 15.25) goodness-of-fit reported as R^2^, assessed for significance at a 95% fit. These were plotted alongside the South African water quality guideline’s chemical and microbiological limits for application in monitoring potable water storage [17]. National guidelines are based on the World Health Organization guidelines [18], and comparable to those of leading countries like Canada [19].

#### Chemical profile of river water samples

Chemical Oxygen Demand (Spectroquant COD Cell Test, 1.14541.0001), Nitrogen (Spectroquant Nitrogen (total N) Cell Test, 1.00613.0001), Ammonium (Spectroquant Ammonium NH_4_^+^ Test, 1.14752.0001), Sulphate (Spectroquant Sulfate SO_4_^2-^ Cell Test, 1.14548.0001) and Phosphate (Spectroquant Phosphate Test, 1.14842.0001) were measured colorimetrically using a Merck Spectroquant Pharo300 photometer, according to the manufacturer’s instructions. Samples were heated in a Spectroquant TR320 Thermocycler, thoroughly mixed by inversion before analyzing and filtered with cellulose nitrate, 1.2 μm pore size filters (Sartorius Stedim Biotech, South Africa). Spectroquant kits and apparatus were sourced from Merck (Modderfontein, South Africa). Correlations between chemical profiles and headspace detection of CO_2_ were determined in triplicate against linear regression models in Microsoft Excel for Mac (v 15.25), and goodness-of-fit reported as R^2^, assessed for significance at a 95% fit.

#### Microbiological profile of river water samples

Standard Heterotrophic Plate Counts were conducted (APHA Method 9215) on Standard Plate Count media (Yeast Extract, 2.5 g/L; Pancreatic Digest of Casein, 5.0 g; Glucose, 1.0 g/L; Agar, 15 g/L; pH 7.0; 26°C). Total coliform counts were assessed on EndoAgar (Merck, APHA Filtration Method 9222; 37°C). Gram negative enteric bacteria were assessed on MacConkey Agar (APHA Filtration Method 9222; 37°C). In addition to filtration, where bacterial loads were higher, reactor samples were diluted in Physiological Saline Solution (9 g/L NaCl), and enumerated on Standard Plate Count, Endo and MacConkey Agar. Chemicals were purchased from Sigma-Aldrich (Johannesburg, South Africa). Correlations between microbiological profiles and headspace detection of CO_2_ were determined in triplicate against linear regression models in Microsoft Excel for Mac (v 15.25), and goodness-of-fit reported as R^2^, assessed for significance at a 95% fit.

#### ATP measurements

ATP [Relative Light Units (RLU)/50 μL] was measured as a confirmation of metabolic profiles. River water was added (50 μL) to Hygenia Ultrasnap ATP surface swabs (Fischer Scientific, Ottawa, Canada), shaken for 15 s and measured in a Hygenia EnSURE luminometer according to manufacturer’s instructions. These are used to measure microbial surface contamination in the food and hospital industry [20], but were harnessed here as a simple tool for confirming metabolic activity in a river water dilution series. Dispersion of flocs was critical for unbiased readings. Samples (1.5 mL) were vortexed (2 minutes) prior to analysis. Correlations between metabolic profiles and headspace detection of CO_2_ were determined in triplicate against linear regression models in Microsoft Excel for Mac (v 15.25), and goodness-of-fit reported as R^2^, assessed for significance at a 95% fit.

#### Closed-loop design

To investigate lower detection thresholds, the system was redesigned to accumulate CO_2_ over time (Fig 1 –closed loop), by circulating the reactor headspace continuously over the CO_2_ analyzer. An overnight culture of *E*. *coli* (3 g/L Tryptic Soy Broth, TSB, 26 °C, rotary shaker) was diluted (sterile tap water) and inoculated into a 5 L Erlenmeyer flask containing autoclaved tap water, at final concentrations of 10^1^, 10^2^ and 10^3^ CFU/100 mL, with continuous stirring. Overnight *E*. *coli* concentrations were pre-determined with growth curves at room temperature rather than 37 °C in TSB, to prevent temperature variation upon transfer. Final cell concentrations were checked by extraction from the sampling port, and counted on Tryptic Soy Agar using filtration (TSA, 3 g/L TSB, 15 g/L agar), as described above for total heterotrophic analysis. The accumulation of CO_2_ over time was measured at each cell concentration, before the cell concentration was increased via the injection port.

### Canary CEMS for active bioreactor disturbances

For RAS bioreactors, the flasks were filled (4.5 L) with synthetic wastewater and sterilized by autoclaving. The reactors were inoculated with 0.5 L activated sludge, corresponding to a liquid volume of 5L and a headspace volume of 0.9 L, and allowed to equilibrate to steady state CO_2_ production with constant stirring. One of the two reactors was sterilized in the autoclave after the addition of RAS and maintained under identical conditions as an abiotic control. The reactors were treated with identical environmental stressors. These chemical and physical disturbances included (1) chlorine [ChlorGuard commercial bleach; final reactor concentration 15% (v/v)], (2) acidification [(a) pH 6.6 dropped to pH 5.0, ~200 μL concentrated (38%) HCl and (b) pH 6.6 dropped to pH 3.3, ~600 μL concentrated HCl)] and (3) temperature fluctuations [23°C dropped to 16°C; 5% (v/v) ice, in a 4 °C refrigerator]. The chlorine treatment was repeated, once with reactor mixing (aerated) and once without (sedimentary). For the pH disturbances, soluble CO_2_ was measured pre- and post-acidification with a MerckMillipore Carbon Dioxide Titrimetric MCarbon Test (Modderfontein, South Africa), according to manufacturer’s instructions. A Jenway 3510 pH Meter, calibrated using the 3 buffer method, was used to monitor pH. Titrations were performed and data collected using a HI902 Auto-titrator (Hanna Instruments^®^) equipped with a HI1131 pH probe (Hanna Instruments^®^). The instrument was pH calibrated beforehand using standards pH 4, pH 7 and pH 10 (Merck Millipore). Up titrations were performed to endpoint pH 11 using standardized 0.1 N NaOH after which subsequent down titrations were performed with standardized 0.1 N HCl to endpoint pH 5.

For all treatments, cell concentrations were measured pre- and post-disturbance. Standard plate counts were assessed with dilutions on Tryptic Soy Agar (Sigma Aldrich, Johannesburg, South Africa).

#### Mathematical modelling of physico-chemical and microbiological CO_2_ release

A dynamic mathematical model (the details of which can be found in S1 Appendix) was used to discriminate between physico-chemical- and microbiological CO_2_ release from the liquid. A fundamental assumption of this model was that the microbiological CO_2_ production was constant. While this assumption may appear limiting, it has little effect on the results interpretation, as demonstrated shortly.

The model accounted for microbiologically produced CO_2_, speciation into bicarbonate (HCO_3_^-^ and carbonate CO_3_^2-^), and CO_2_ mass transfer between the liquid- and gas phases. The model was implemented using MATLAB R2018a (The MathWorks, Inc., Natick, MA).

All model parameters are provided in Table A1 in S1 Appendix. Two parameters were unknown: the microbiological CO_2_ production rate (${\dot{r}}_{CO2}$) and the liquid-air mass transfer coefficient (*K*_*L*_*A*). Both were determined by regression of experimentally measured gaseous CO_2_ concentrations to model predictions.

#### Threshold alarm software

Remote alarm functionality was achieved by developing an external software application in the Python programming language to intercept the data packets on the LiCor LI-820 CO_2_ analyzer’s serial port. This allowed users to specify an upper and lower threshold, and to configure an email message, sent when serial port readings surpassed any of the pre-determined thresholds.

## Results

The measurement of microbial CO_2_ production for the remote monitoring of water treatment system performance was assessed. Two experimental scenarios were evaluated, on the polar ends of a metabolic spectrum: (a) for the remote monitoring of microbial contamination in potable water systems (where the ideal microbial levels are zero), and (b) for the remote monitoring of steady state microbial activity in bioreactors for waste treatment (where steady-state CO_2_ production is optimal within a pre-determined window).

### Headspace volume and detection sensitivity

**Fig 2:**
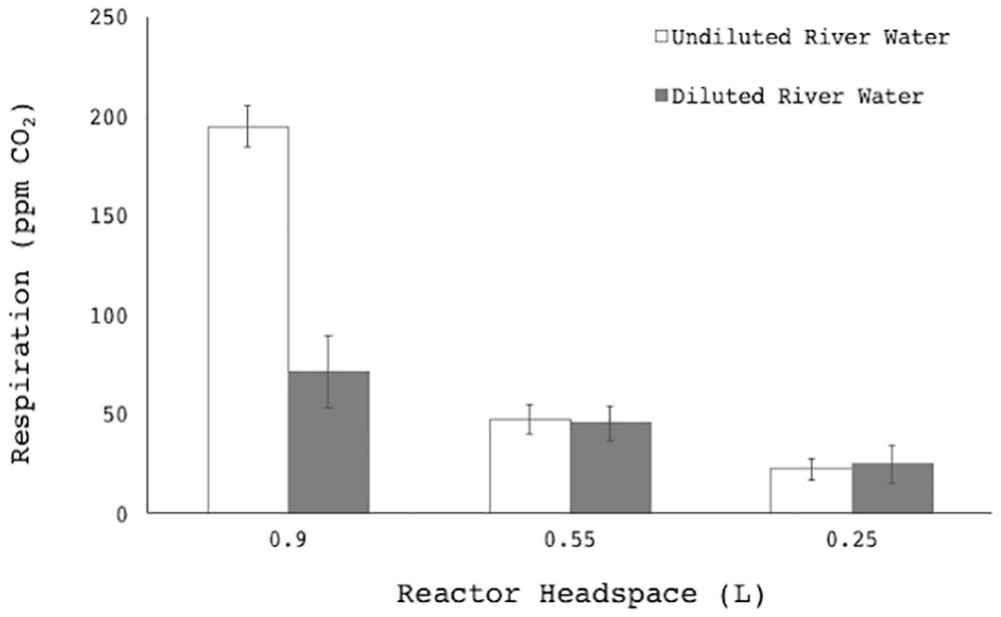
The effect of reactor volume and headspace on (a) the whole-system CO_2_ measurement. The first reactor (1) contained undiluted river water at (A) 5 L with 0.9 L headspace, (B) 5.35 L with 0.55 L headspace, and (C) 5.65 L with 0.25 L headspace. The second reactor (2) contained diluted Plankenbrug river water (25% v/v sterile tap water), at the same volume:headspace ratios. Controls were a separate reactor with undiluted autoclaved Plankenbrug river water. Respiration was compared by subtracting the average CO_2_ production of the sterile control reactor from the average CO_2_ production of the reactor over a given time period. Error bars represent standard deviation of measurements per second over 1 hour.

Respiration was monitored in reactor headspaces, which were open to the atmosphere via a defined inlet port. Baselines were predictable at 385 ppm CO_2_ ± 45 ppm for the open-loop system, with variation attributed to human activity in the vicinity increasing CO_2_ levels during the day. Closed-loop baselines had a similar standard deviation if measured at separate instances over different days, but a variation of less than 5 ppm over days if sealed. Headspace CO_2_ was allowed to accumulate before open-loop measurement. In river water reactor systems, a smaller headspace decreased the sensitivity of the whole-system CO_2_ production (Fig 2, Student’s t-Test, p<0.05). This was counter-intuitive, as lower headspace volumes are correlated with greater river water volumes, increasing the total biomass and thus presumably resulting in increased CO_2_ production. These unexpected results are likely due to two reasons: (1) the system is open to ambient air and a smaller headspace is more rapidly replaced by ambient air drawn into the system, and (2) the conical flasks in the laboratory set-up meant a larger headspace was equivalent to a larger surface area for gas-liquid CO_2_ exchange. The tension between the influences of these design parameters on the data indicates that case-by-case optimization is necessary for effective industrial CEMS application as an early-warning alarm system for microbial disturbances in water storage systems.

**Fig 3:**
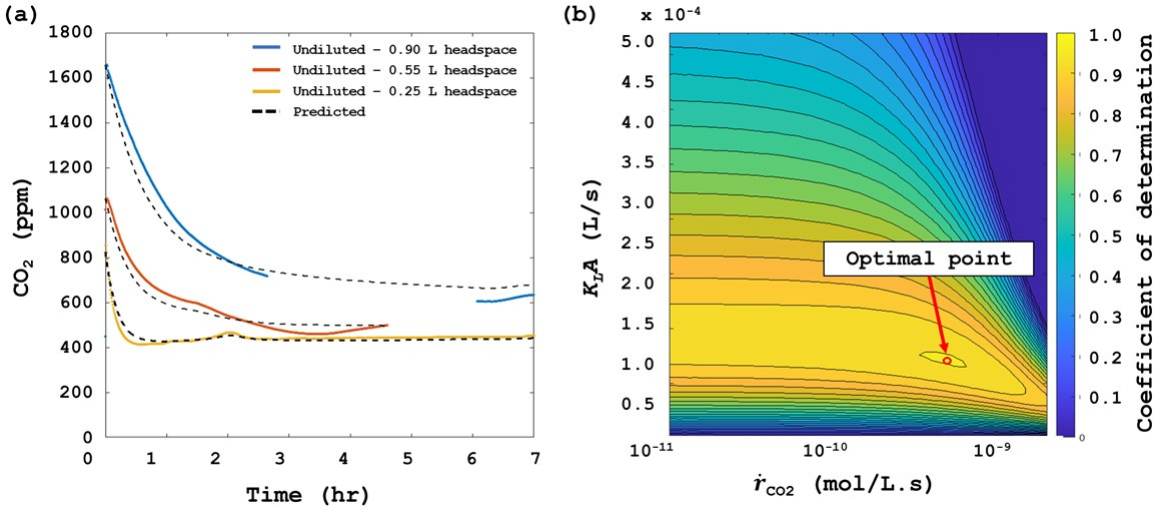
Modelling of CO_2_ limits of detection. (a) Predicted vs measured values of CO_2_ partial pressure in the headspace above undiluted river water for varying headspace volumes. CO_2_ was allowed to accumulate in the headspace prior to turning on the CEMS. (b) Residual Sum of Squares as a function of the regressed parameters r˙_CO2_ and K_L_A. At low contamination levels, the rate of CO_2_ production can vary by multiple orders of magnitude without significantly affecting the measured response, thereby indicating the CEMS limits of detection.

The dynamic model results compare well to the experimentally measured CO_2_ partial pressure (Fig 3a), but the rate of CO_2_ production (as determined by regression) was so low in the river water that the regression was insensitive to orders of magnitude variations in ${\dot{r}}_{CO2}$ in the open-loop configuration, which is to be expected with only a few cells responsible for the metabolic footprint. Fig 3b shows the variation in the coefficient of determination (1-TSS/RSS, where TSS is the total sum of square errors from the mean, and RSS is the residual sum of squares) as a function of the regressed parameters *K*_*L*_*A* and ${\dot{r}}_{CO2}$. Clearly, the values for ${\dot{r}}_{CO2}$ can vary significantly without influencing the fraction of explained variance. This implies that the CEMS device is not suitable for such low CO_2_ production rates in an open-loop design. However, the combination of modelling and experimentation as performed in this study can be used to indicate the range at which the system can be used, guiding further modification of the system into a closed-loop design as described below.

The open-loop CEMS data proved insufficient to accurately predict the rate of CO_2_ production in systems with low levels of microbial contamination. However, the steady state CO_2_ concentrations in such systems were still significantly higher than in their abiotic counterparts. This implies that the steady state CO_2_ concentration as detected by the CEMS can be used as an empirical predictor of water quality. Given the enhanced sensitivity at larger headspace volumes, correlating to larger surface area, a liquid volume of 5 L (corresponding to a headspace volume of 0.90 L) was used for all subsequent experiments.

### Monitoring microbial contamination: Potable water systems

Elevated CO_2_ concentrations in the reactor headspace was measurable for undiluted polluted river water in an open system. In order to determine the sensitivity thresholds of the system, the river water was diluted (50%, 25%, 10%, 1%, 0% v/v) with sterile tap water, representing an increasingly contaminated water storage system, and the profiles were assessed at each dilution.

- chemical (COD, Total-N, NH_4_^+^-N, SO_4_^-^),
- microbiological (standard heterotrophic bacteria, total coliforms, enteric bacteria), and
- metabolic (CO_2_, ATP)

**Fig 4:**
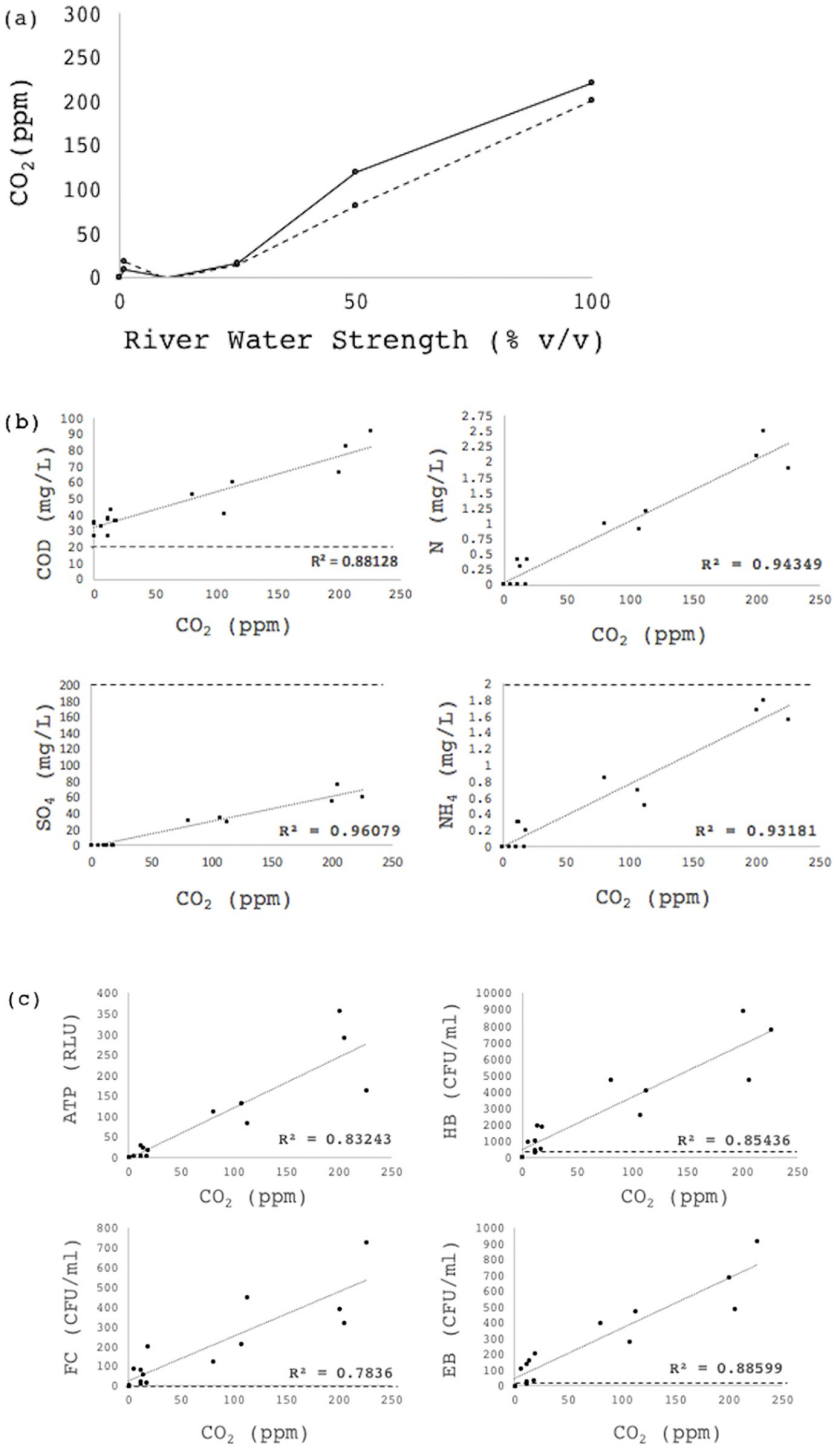
(a) Pollutant effects on CO_2_ measurements. CO_2_ profiles for increasing river water concentrations (pollution levels) were plotted for 2 independent sampling events. (b) Pollutant effects on CO_2_ measurements. River water chemistry was correlated against CO_2_ at the above-mentioned dilutions, including COD (mg/L), sulphate (mg-SO4-/L), total nitrogen (mg-N/L) and ammonia (mg-NH4+/L). The results of triplicate experiments are plotted. Minimum thresholds are indicated with dashed lines, as determined by the South African Guidelines for Drinking Water Standards [17]. (c) River water energetic (ATP) and microbiological profiles were correlated against CO_2_ at the above-mentioned dilutions, including fecal coliforms (FC), enteric bacteria (BC) and heterotrophic bacteria (HB). The results of triplicate experiments are plotted. Minimum thresholds are indicated in red, as determined by the South African Guidelines for Drinking Water Standards [17].

The dilution profile of polluted river water produced a measurable CO_2_ production trend, which was correlated to a suite of water quality parameters, described in the national water quality guidelines [17]. Adenosine triphosphate (ATP) was included as an independent measure of metabolic activity, detected with fluorometry on swabs typically utilized in surface metabolic monitoring. This technique confirmed the metabolic profile with tight correlation to CO_2_ data (R^2^ = 0.832; Fig 4b), but generated high variation at higher bacterial loads, likely due to challenges in microbial floc dispersion. All of the chemical and microbiological parameters also correlated with CO_2_ production (Fig 4b and 4c), and the point at which the detection system’s correlation with the chemical parameters (total N, NH_4_-N and sulphate) was useful fell above the minimum thresholds according to South African Water Quality Guidelines [17]. In contrast, the limits prescribed by these guidelines for COD and the microbial loads were well below the lowest threshold of the system. The correlation (R^2^ = 0.88; Fig 4) between enteric bacterial load and CO_2_ production indicated promising potential for detection of larger contamination events. However, the target water quality range for total heterotrophs, according to SANS241 [17], is 0–100 CFU/mL, which is below the lowest threshold of the CO_2_ correlation in open-loop configuration, and the target water quality range for coliforms is drastically more stringent at 0–5 CFU/100 mL.

**Fig 5:**
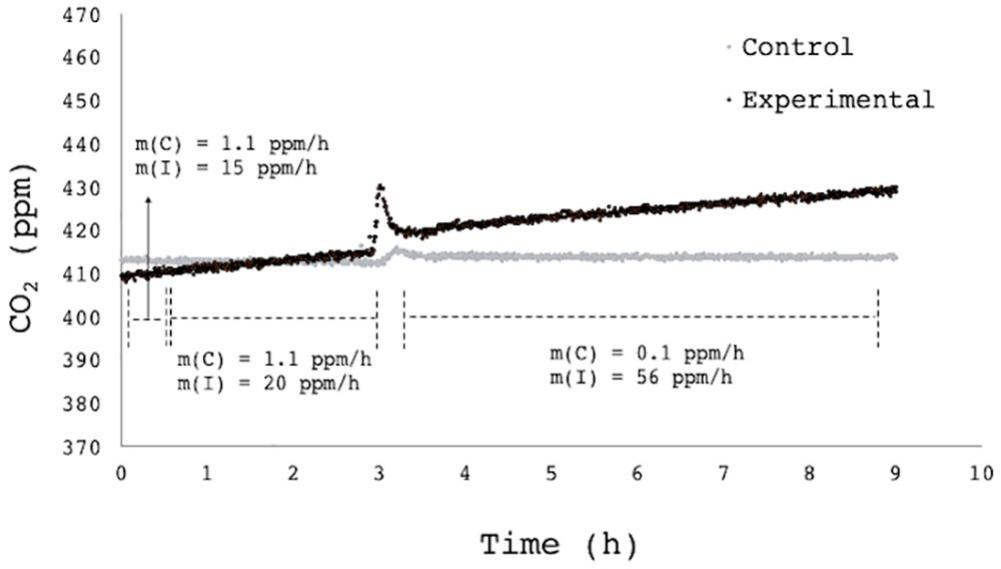
Headspace accumulation of CO2 in in a closed-loop CEMS design. A control (C) reactor containing sterile water was compared to *E*. *coli* inoculated (I) reactors. The inoculation concentration was increased twice: from an initial concentration of 10 CFU/mL over time range (A), it was increased to 100 CFU/mL for time (B) and finally (C) 1000 CFU/mL. The CO_2_ generation rates m(C) and m(I) in the control and inoculated reactors, respectively, are indicated in the figure, clearly showing a detectable difference in each case.

Based on these threshold limitations of the open-loop CEMS alarm system, measuring CO_2_ production on a continuously replaced carrier gas, the design was adapted to measure closed-loop CO_2_ accumulation, in an attempt to decrease the lower threshold (Fig 1). It was only assessed against the microbial load, which is the limiting parameter (Fig 4). In this case, the rate of CO_2_ accumulation, calculated from the slopes, was compared (ppm/h) at 10 CFU/100 mL, 10^2^ CFU/100 mL and 10^3^ CFU/100 mL (total coliforms). Here, the CO_2_ accumulation rates were statistically distinguishable as low as 10 CFU/100mL according to linear best fit models, however the tool is truly useful over 100 cfu/100 mL, as the slopes at 10 and 100 cfu/mL only differ by 5 ppm, and control variation is 1.8 ppm (Fig 5). An automated flushing mechanism to facilitate closed-loop daily monitoring has been designed in-house, to allow for a pre-determined window of CO_2_ accumulation before flushing, circumventing perpetual CO_2_ accumulation.

It is clear from these thresholds that the open-loop Canary CEMS is an indicator of significant, sudden contamination events, but is not suited for the monitoring of drinking water to potable national and international standards. The lack of suitability is based largely on sensitivity of the system for measuring microbial activity at levels as low as guidelines demand, which is effectively zero. The alarm effectively detects microbial loads higher than 10^2^−10^3^ CFU/mL, above the maximum thresholds for potable water. In attempting to compare the detection range to other widely promoted measurements techniques, such as qPCR, the definition of Limits of Quantification (LoQ’s) are informative, including precision and accuracy, only under *stated experimental conditions* [21]. Forootan et al. [22] explore the challenges of defining thresholds of detection and quantification of qPCR. In their context, the LoD (Limit of Detection) was 2 molecules, and the LoQ was 16 molecules. This is tenfold lower than the CEMS alarm, but still demands time, laboratory facilities and expertise.

Contamination events that have warranted reporting due to associated public illness include ranges as wide as 20 CFU/mL [23] to 10300 CFU/mL [24]. This work demonstrates the utility of the technology as an early warning system for such significant contamination events, as well as for applications in aquaculture, irrigation (water quality limited to 100 CFU/mL, according to South African National Guidelines [25]) and service water for washing equipment.

### Monitoring bioreactor disturbances

**Fig 6:**
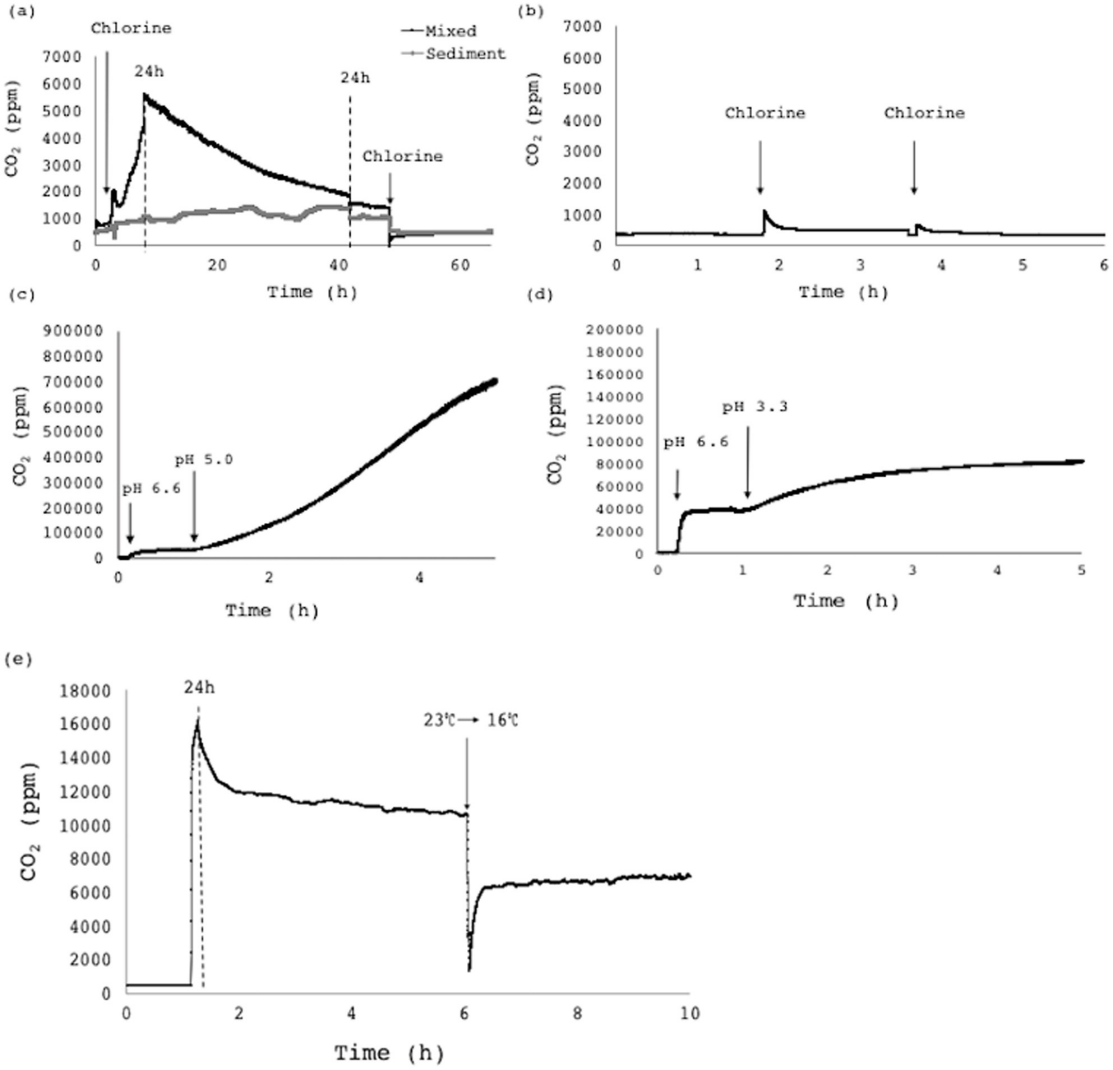
CO_2_ fluctuations in response to physico-chemical disturbances. The CO_2_ profiles of (a) a mixed and sedimentary RAS reactor with the addition of chlorine compared to (b) an autoclave sterilized RAS control reactor; as well as a mixed RAS reactor acidified from pH 6.6 to (c) 5.0 and (d) 3.3, with concentrated HCl, and (e) a sudden cooling event (ice; ~5% v/v) in a mixed RAS reactor connected to the system at 1 h. Measurement intervals are indicated with dashed lines.

The alarm system was proven effective as an indicator of microbial inhibition and metabolic disturbances in active return activated sludge (RAS) reactors. In the case of a disinfectant (chlorine, Fig 6a and 6b), a pH disturbance (pH drop from 6.6 to 5.0 and 3.3, HCl, Fig 6c & 6d respectively) and a temperature disturbance (temperature decrease: 22°C to 16°C, Fig 6e), the CO_2_ immediately fluctuated well outside of pre-determined windows defined by steady-state CO_2_ measurements.

The first disinfectant treatment triggered an approximately 5-fold increase in the metabolic activity of a mixed reactor (Fig 6a). The CO_2_ measurements subsequently steadily decreased over days, remaining almost 2-fold higher than the pre-treated whole-reactor CO_2_ production rate. The control reactor showed a slight increase in CO_2_ production upon addition of chlorine, probably due to chemical oxidation of organic material in the water, although the metabolic measurements were negligible in comparison to the non-sterile reactor confirming the biological metabolic response (Fig 6b). The sedimentary reactor demonstrated a similar, although less dramatic, response upon disinfectant treatment, suggesting a higher sedimentary chlorine demand. A second treatment with the same disinfectant concentration dropped the measurable CO_2_ production rate to zero for both the mixed and sedimentary reactors, and cell survival was also approximately zero after the second chlorine treatment. It is well established that microbes respond to stressors such as antibiotic challenges with a spike in CO_2_ production, likely due to the metabolic shifts for adaptation [7, 8], particularly with continued exposure to the chemical inhibitor. Higher CO_2_ production is potentially ascribed to the metabolic cost associated with the upregulation of efflux pump activity in the cell [7]. This work indicates a similar metabolic response to an oxidizing agent (Fig 6a).

Similarly, a pH decrease from 6.6 caused the microbial consortium to increase its metabolic rate (Fig 6c and 6d). The measurable metabolic rate was approximately 10-fold higher at pH 5.0 than at pH 3.3. Acidification to pH 3.3 caused a drop in cell concentration from 10^9^ CFU/mL to 10^7^ CFU/mL in 2 h and to 10^3^ CFU/mL in 24 h, whereas cell concentrations remained constant at 10^9^ CFU/mL with a milder pH drop to 5.5. The 10-fold difference in CO_2_ production is due to an interaction between this difference in biological contribution, as well as physico-chemical CO_2_ speciation.

Temperature and pH affect the liquid phase speciation of CO_2_ (see S1 Appendix) and will affect the liquid-to-headspace mass transfer rate. A decrease in pH shifts the carbonate equilibria from ionic bicarbonate towards dissolved CO_2_, which would then escape to the atmosphere and subsequently increase the headspace CO_2_ concentration. Assuming the liquid- and gas phases are in equilibrium and that the acidification process is rapid enough such that the total (combined liquid phase and headspace) molar amount of CO_2_ in the reactor remains constant, it is shown that the proportional increase in CO_2_ partial pressure *p*_*CO*2_ is given by Eq 1 (subscripts 0 and *f* indicate reactor conditions before and after acidification, see S1 Appendix):

$$\frac{p_{CO2,f}}{p_{CO2,0}}=\frac{\frac{V_{G}}{RT_{0}}+\frac{V_{L}}{K_{H}(T_{0})}(1+\frac{K_{1}(T_{0})}{10^{-pH_{0}}}+\frac{K_{1}(T_{0})K_{2}(T_{0})}{10^{-2pH_{0}}})}{\frac{V_{G}}{RT_{f}}+\frac{V_{L}}{K_{H}(T_{f})}(1+\frac{K_{1}(T_{f})}{10^{-pH_{f}}}+\frac{K_{1}(T_{f})K_{2}(T_{f})}{10^{-2pH_{f}}})}$$ (1)

Where *V*_*G*_ and *V*_*L*_ are the gas- and liquid-phase volumes, *R* is the universal gas constant, and *K*_*H*_, *K*_1_ and *K*_2_ are the Henry’s constant and dissociation constants of dissolved CO_2_. The equilibrium constants are all functions of temperature.

Within this study, the ratio of steady state CO_2_ partial pressures (before and after acidification) due to purely chemical effects, calculated using Eq 1, is compared to the ratio of steady state CO_2_ partial pressures as measured using the CEMS (Table 1). When the pH was dropped to a value of 5.0 from pH 6.6, the measured 24-fold increase in CO_2_ partial pressure far exceeded the values solely attributed to a disturbance in the chemical equilibrium, thus indicating that acidification triggered a metabolic response. In the case of acidification to pH 3.3, the measured 2.3-fold increase in CO_2_ partial pressure was slightly lower compared to that calculated for a purely chemical response. This indicates a complete cessation of metabolic activity. The lower than expected increase in CO_2_ partial pressure can be attributed to the continuous removal of CO_2_ from the reactor headspace by the CEMS.

**Table 1:** Ratio of CO_2_ partial pressures before and after acidification due to purely chemical effects (calculated) compared to combined chemical and metabolic effects (measured).

Initial pH	Final pH	$\frac{p_{CO2,f}}{p_{CO2,i}}\;(calculated)$	$\frac{p_{CO2,f}}{p_{CO2,i}}\;(measured)$
6.6	5.0	2.5	23.9
6.6	3.3	2.7	2.3

In contrast, temperature fluctuations did not lead to a spike in CO_2_ production rates, but rather a drop in respiration to approximately 65% upon cooling (Fig 6e). There was no associated decrease in microbial load with the temperature fluctuation. The phenomenon of decreased respiration with decreasing temperature is well-demonstrated in soil microbial communities [26].

The temperature decrease, from 22 °C to 16 °C, would affect the chemical equilibrium mathematically represented by *K*_*H*_, *K*_1_ and *K*_2_. In this case, the CO_2_ partial pressure due to chemical effects alone was calculated to be 10%, underestimating the measured decrease of 40%, clearly indicating a decrease in metabolic activity.

The LiCor CO_2_ monitoring system software was also adapted to send an email in response to CO_2_ fluctuations outside pre-determined thresholds, alerting the system manager of metabolic disturbances, remotely and immediately. This adaptation eliminates the need for expertise to assess microbial activity, and the consequent performance of the bioreactor. It makes it accessible to technicians, which is critical in implementing this technology, particularly in rural, under-developed areas. Design adjustments might involve the circulation of a representative reactor sample through an adjacent compartment controlled for volume and headspace, rather than direct measurement of the reactor headspace, as well as the measurement of biofilms in pipes.

There is no silver bullet for the monitoring of water storage contamination events, be it potable, irrigation or water recirculation for cleaning—a common practice in drought-ridden areas. The high frequency of water quality system collapse demands creative additions to the current suite of water-monitoring techniques. There is a tension between the resolution of microbial water quality information, the cost and the speed at which information is accessible.

Thus, the efficiency of this technology can be compared to competing technology [27]. Such competing technologies, based on fluorescence assays and enzyme detection, still involve grab samples, and demand technical know-how and complex, energy-expensive technology. However, the true benefit of this work lies not in competition, but rather in the amplified benefits of multipronged approaches to water quality monitoring. The cumulative effect of both high-level, remote, online alarm systems and more detailed pathogen identification, will provide more effective protection to communities susceptible to outbreaks. In addition, the particular benefit of this technology is the accessibility to under-developed areas without geographical and economic access to standard laboratory facilities: often the most vulnerable communities.

## Conclusions

The Carbon Dioxide Evolution Monitoring System was shown to only be indicative of relatively high levels of microbial contamination in water systems. The lower thresholds are above the maximum coliform and heterotrophic loads permitted for potable drinking water according to WHO and national standards, although significantly improved by the redesign to a closed-loop system. In contrast to potable water standards, it was proven effective for significant contamination events and for monitoring water for broader applications, for example to national and international irrigation standards. The canary CEMS was also effective as an immediate, remote alarm for metabolic disturbances in a RAS reactor treated with a disinfectant, as well as pH and temperature fluctuations. The aim of the system is to act as an immediate indicator of the metabolic responses of the microbial aggregate, which was resolved from the physico-chemical CO_2_ equilibration by mathematical modelling. If thresholds and minimum limits are understood and optimized for individual application per system, the universal principle of whole microbiome respiration has notable potential as an early-warning technology for microbial disturbances in industrial settings.

## Supporting information

S1 Appendix Mathematical model of CO_2_ release. (DOCX)
