# Supplementary material for: Canary in the coliform mine: Exploring the industrial application limits of a microbial respiration alarm system
Source: PLoS One. 2021 Mar 4;16(3):e0247910. doi: 10.1371/journal.pone.0247910 (PMC7932117; doi:10.1371/journal.pone.0247910)
Supplement: S1 Appendix — (DOCX) [file pone.0247910.s001.docx]

**S1 Appendix. Mathematical model of CO_2_ release**

The volumetric rate of CO_2_ production ($\dot{r}_{CO2}$) was estimated by modelling the CEMS system and finding the (assumed constant) $\dot{r}_{CO2}$ value that resulted in the lowest Residual Sum of Squares (RSS) between the predicted ($p_{CO2}$) and measured ($p_{CO2,m}$) values at each measured time point $t_{j}$ (eq. A1):

| $\dot{r}_{CO2}=\min_{\dot{r}_{CO2}\geq0} \sum_{j} \left( p_{CO2}\left( t_{j} \right)-p_{CO2,m}\left( t_{j} \right) \right)^{2}$ |  |
| --- | --- |

The CO_2_ partial pressure was modelled by considering a CO_2_ balance over the liquid- and headspace- volumes of the CEMS reactor (eqns. A2-A3):

| $V_{L}\frac{dc_{T}}{dt}=-\dot{n}_{lg}+\dot{r}_{CO2}V_{L}$ |  |
| --- | --- |
| $\frac{V_{G}}{RT}\frac{dp_{CO2}}{dt}=\dot{n}_{atm}\frac{p_{CO2,atm}}{P}-\dot{n}_{F}\frac{p_{CO2}}{P}+\dot{n}_{lg}$ |  |

Where $V_{L}$ and $V_{G}$ are the liquid and headspace volumes, respectively, while $T,P,p_{CO2,atm}$ and $R$ are the temperature, pressure, atmospheric CO_2_ partial pressure and the universal gas constant. The molar flowrate through the CEMS device ($\dot{n}_{F}$), through the port that was open to the atmosphere ($\dot{n}_{atm}$) and from the liquid- to the gas-phase ($\dot{n}_{lg}$) are related by a molar balance over the headspace volume (eq. A4), assuming a constant pressure:

| $\dot{n}_{atm}+\dot{n}_{lg}=\dot{n}_{F}=\frac{P\dot{F}}{RT}$ |  |
| --- | --- |

The molar flowrate through the CEMS device is proportional to the volumetric flowrate $\dot{F}$ through the same, which was held constant at 0.015 L/min. The flowrate from the gas to the liquid phase due to mass transfer is given by eq. A5:

| $\dot{n}_{lg}=K_{L}A\left( c_{CO_{2}}-\frac{p_{CO2}}{K_{H}} \right)$ |  |
| --- | --- |

Where $K_{L}A$ represents the gas-liquid mass transfer coefficient, $c_{CO_{2}}$ is the concentration of dissolved CO_2_ and $K_{H}$ is the Henry’s law constant. Dissolved CO_2_ can react with water to form carbonic acid and dissociate into bicarbonate and carbonate (eq. A6). The speciation equilibrium is given by eqns. A7 and A8:

| $H_{2}O+CO_{2}\left( \mathrm{aq} \right)\rightleftharpoons H_{2}CO_{3}\left( \mathrm{aq} \right)\rightleftharpoons\mathrm{HC}O_{3}^{-}\left( \mathrm{aq} \right)+H^{+}\rightleftharpoons CO_{3}^{2-}\left( \mathrm{aq} \right)+2H^{+}$ |  |
| --- | --- |
| $K_{1}=\frac{c_{HCO_{3}^{-}}c_{H^{+}}}{c_{H_{2}CO_{3}^{*}}}\approx\frac{c_{HCO_{3}^{-}}c_{H^{+}}}{c_{CO_{2}}}$ |  |
| $K_{2}=\frac{c_{CO_{3}^{2-}}c_{H^{+}}}{c_{HCO_{3}^{-}}}$ |  |

Note that eq. A7 is written in terms of the combined concentration $c_{H_{2}CO_{3}^{*}}=c_{CO_{2}}+c_{H_{2}CO_{3}}$. Given that $c_{CO2}/c_{H_{2}CO_{3}}$ $\approx1000$ at equilibrium, the simplification $c_{CO_{2}}\approx c_{H_{2}CO_{3}^{*}}$ was used throughout. The total dissolved carbon concentration (as per eq. A2) is given by eq. A9:

| $c_{T}=c_{CO2}+c_{HCO_{3}^{-}}+c_{CO_{3}^{2-}}$ |  |
| --- | --- |

Finally, the hydronium ion concentration $c_{H^{+}}$ can be estimated using a charge balance (eq. A10):

| $c_{ALK}+c_{H^{+}}=c_{OH^{-}}+c_{HCO_{3}^{-}}+2c_{CO_{3}^{2-}}$ |  |
| --- | --- |

Where $c_{ALK}$ is the total alkalinity and $c_{OH^{-}}$ is the hydroxide ion concentration, calculated using the dissociation constant of water (eq. A11):

| $c_{H}^{+}c_{OH}^{-}=K_{W}$ |  |
| --- | --- |

Equations A2-A11 represents a closed system of differential-algebraic equations (DAE) which can be solved using a numerical integrator. MATLAB R2018a (The MathWorks, Inc., Natick, MA) was used to solve the DAE system. All parameters are listed in Table A1. Three system specific, unknown parameters remain: $\dot{r}_{CO2}, K_{L}A$ and $c_{ALK}$. The alkalinity can be estimated using the measured initial pH and assuming that the liquid- and gas-phases are in equilibrium before the CEMS is turned on. The remaining two parameters ($\dot{r}_{CO2}$ and $K_{L}A$) are determined by minimizing the RSS. The values obtained by regression are also given in Table A1.

**Table A1. Parameters used in the dynamic model of CO_2_ evolution.** Temperature dependent correlations for $K_{H}, K_{1}, K_{2}$ and $K_{W}$ are provided by Plummer and Busenberg [1] and Smith, Kim and Lineberger [2].

| **Parameter** | **Value** | **Description** |
| --- | --- | --- |
| $\boldsymbol{V}_{\boldsymbol{L}}$ | 5.0 L  5.25 L  5.65 L | Liquid volume  Experiments A, B and C |
| $\boldsymbol{V}_{\boldsymbol{G}}$ | 0.90 x 10^-3^ m^3^  0.55 x 10^-3^ m^3^  0.25 x 10^-3^ m^3^ | Headspace volume  Experiments A, B and C |
| $\boldsymbol{R}$ | 8.314 Pa.m^3^.mol^-1^.K^-1^ | Universal gas constant |
| $\boldsymbol{T}$ | 296 K | Ambient temperature |
| $\boldsymbol{P}$ | 101 325 Pa | Ambient pressure |
| $\boldsymbol{p}_{\boldsymbol{CO2,atm}}\boldsymbol{(t)}$ | Varies with time (Pa) | Measured over the course of each experiment |
| $\dot{\boldsymbol{F}}$ | 15 mL.min^-1^ = 2.5 x 10^-7^ m^3^.s^-1^ | Volumetric flowrate through CEMS |
| $\boldsymbol{K}_{\boldsymbol{H}}$ | 2.94 x 10^6^ Pa.L.mol^-1^ | Henry’s constant for CO_2_ |
| $\boldsymbol{K}_{\boldsymbol{1}}$ | 10^-6.3^ | Carbonic acid first dissociation constant  (using $c_{H_{2}CO_{3}}^{*}$) |
| $\boldsymbol{K}_{\boldsymbol{2}}$ | 10^-10.32^ | Carbonic acid second dissociation constant |
| $\boldsymbol{K}_{\boldsymbol{W}}$ | 10^-14^ | Dissociation of water |
| $\boldsymbol{c}_{\boldsymbol{ALK}}$ | 0.0026 mol.L^-1^  0.0017 mol.L^-1^  0.0013 mol.L^-1^ | Estimated using initial values |
| ${\dot{\boldsymbol{r}}}_{\boldsymbol{C}\boldsymbol{O}_{\boldsymbol{2}}}$ | 4.3 x 10^-10^ mol.L^-1^.s^-1^ | Regressed |
| $\boldsymbol{K}_{\boldsymbol{L}}\boldsymbol{A}$ | 1.5 x 10^-4^ L.s^-1^ | Regressed |

Many metabolic shocks (e.g. pH and temperature) affect the carbon dioxide-bicarbonate equilibrium shown in eq. A6 and will affect the rate of mass transfer from the liquid to the headspace. If the CEMS is to be used as an alarm system, it must be able to distinguish between changes in CO_2_ resulting from chemical or biological sources.

The total molar amount of carbon dioxide contained in the reactor head space ($n_{G}$) and liquid phase ($n_{L}$), at equilibrium, is given by summing over all possible species and substituting eqns. A7 and A8 as well as Henry’s law ($c_{CO2}=p_{CO2}/K_{H}$) to eliminate the aqueous species terms as shown in eq. A12:

| $n_{G}+n_{L}=\frac{p_{CO2}V_{G}}{RT}+V_{L}\left( c_{CO2}+c_{HCO_{3}^{-}}+c_{CO_{3}^{2-}} \right)$ $=\frac{p_{CO2}V_{G}}{RT}+V_{L}c_{CO2}\left( 1+\frac{K_{1}}{c_{H^{+}}}+\frac{K_{1}K_{2}}{c_{H^{+}}^{2}} \right)$ $=p_{CO2}\left[ \frac{V_{G}}{RT}+\frac{V_{L}}{K_{H}}\left( 1+\frac{K_{1}}{c_{H^{+}}}+\frac{K_{1}K_{2}}{c_{H^{+}}^{2}} \right) \right]$ |  |
| --- | --- |

The total molar amount of carbon dioxide depends on the pH through the $c_{H^{+}}$ terms and on temperature through the $K_{1}$, $K_{2}$ and $K_{H}$ terms [28]. Assuming the liquid- and gas phases are in equilibrium and that the physico-chemical processes are rapid enough such that the total (combined liquid phase and headspace) molar amount of CO_2_ in the reactor remain constant before and after a metabolic shock, the proportional increase in CO_2_ partial pressure is given by eq. A13 (where subscripts $0$ and $f$ indicate the reactor conditions before and after a metabolic shock):

| $\frac{p_{CO2,f}}{p_{CO2,0}}=\frac{\frac{V_{G}}{RT}+\frac{V_{L}}{K_{H}}\left( 1+\frac{K_{1}}{{c_{H^{+}}}_{0}}+\frac{K_{1}K_{2}}{{c_{H^{+}}}_{0}^{2}} \right)}{\frac{V_{G}}{RT}+\frac{V_{L}}{K_{H}}\left( 1+\frac{K_{1}}{{c_{H^{+}}}_{f}}+\frac{K_{1}K_{2}}{{c_{H^{+}}}_{f}^{2}} \right)}$ |  |
| --- | --- |

**References**

1. Plummer LN, Busenberg E. The solubilities of calcite, aragonite and vaterite in CO_2_-H_2_O solutions between 0 and 90 C, and an evaluation of the aqueous model for the system CaCO_3_-CO_2_-H_2_O. Geochim Cosmochim Acta. 1982;46(6): 1011-1040.
2. Smith JR, Kim JB, Lineberger WC. High-resolution threshold photodetachment spectroscopy of OH−. Phys Rev A. 1997;55(3): 2036.
