# Supplementary material for: Canary in the coliform mine: Exploring the industrial application limits of a microbial respiration alarm system
Source: PLoS One. 2021 Mar 4;16(3):e0247910. doi: 10.1371/journal.pone.0247910 (PMC7932117; doi:10.1371/journal.pone.0247910)
Supplement: Attachment — Submitted filename: Response to Reviewers.pdf [file pone.0247910.s003.pdf]

Response to Reviewer Comments

The authors would like to thank the reviewers for their continued investment in the article. Each round of revision improves the manuscript notably, and we are grateful for the collaborative and rigorous spirit of the review process.

Please use the space provided to explain your answers to the questions above. You may also include additional comments for the author, including concerns about dual publication, research ethics, or publication ethics. (Please upload your review as an attachment if it exceeds 20,000 characters)

Reviewer #2: The authors present an interesting method for monitoring respiration by quantifying CO2. The device is tested in proof of concept experiments focusing on detection of changes to baseline respiration rates in each system – river water and activate sludge. Overall the data interpretation and modeling need to be improved.

Data and approach – It is unclear why the lumped mass transfer coefficient was fit. This could be evaluated using a control experiment. The potential issue in simultaneously fitting the lumped mass transfer coefficient and the CO2 production rate is that these parameters are likely to be correlated in ways that suggest potential for non-unique optimization/fit. The manuscript describes using R2 for assessing the two parameter fit (r and KLA). It would be helpful to establish that a one parameter model is not capable of describing the data, and then using an information criteria metric (e.g., Akaike) to establish the additional fitting parameter is warranted. A superior approach would be to independently determine KLA (fit to a control test) so as to only fit the rate parameter (i.e., what the device aims to measure) to the experimental data.

We completely agree with the reviewer's comments regarding the simultaneous fit of for the mass transfer coefficient (k_La) and the respiration rate (r_{CO2}). However, this problem is already discussed in Figure 3b, which shows the coefficient of determination (R^2) as a function of k_La and r_{CO2}. The reality is R^2 is largely insensitive to variations in r_{CO2} due to the low rate of respiration. This indicates that, even if the k_La were measured *a priori* using a control experiment, r_{CO2} cannot be subsequently estimated with any reasonable certainty, confirming the reviewer's suspicions. This led us to conclude that the open loop system is inadequate for monitoring surface waters as it lacks the necessary sensitivity. In particular, [lines 348-349]: "The open-loop CEMS data proved insufficient to accurately predict the rate of CO2 production in systems with low levels of microbial contamination." However, the modelling exercise still provided insights into the appropriate setup for subsequent experiments.

What is the implication of the variability in ambient CO2?

The implications of variability will be scenario-specific, in industrial application. The implications are much higher for measuring fluctuations in environments with low metabolic rates. This is a good point, and the motivation to continue with the publication despite the need for scenario-specific research is expounded below. A line has been added in the manuscript acknowledging the implications [Line 299-302].

The manuscript (Line 284) suggests that the baseline is 385 +/-45 ppm. What is the +/- here? The manuscript implies on the next line that it may be a standard deviation, but it is unclear.

The ± is indeed standard deviation, for an average calculated from 10 x 5h baseline measurements, taken at varying times throughout the day (described in Materials and Methods, Line 131-133). We thank the reviewer for pointing out that this was not clearly expressed, and have clarified in the text [Line 295-296].

Since the monitoring device is based on differencing with this ambient condition (Line 127-128), what is the implication on the sensitivity of the motorizing?

We interpret this to be the same question as above, continuing the same thought. The implications of variability will be scenario-specific, in industrial application. The implications are much higher for measuring fluctuations in environments with low metabolic rates. A line has been added in the manuscript acknowledging the implications [Line 295-296].

Later in the manuscript (Line 380-390) there is discussion about detection limit in terms of CFUs. How does the CFU detection limit depend on the substrate and nutrient condition of the water? That is, is using a detection limit in terms of CFUs system dependent? It stands to reason this type of conversion of the detection limit would depend on the rate at which the microbial community can oxidize the carbon. Seemingly this would depend on the form of the carbon present, the type of microbial community, and any limitations on carbon utilization (e.g., carbon, nutrient, and inhibitor/competitor concentrations).

Thank you for this point. It certainly will vary drastically, depending on the nature of the substrate, the metabolic capacity and dynamics of the microbiome, and the environmental conditions influencing the metabolism (as evidenced in simple pH changes and temperature changes in this manuscript). We actually rely on that fact for the success of the technology.

Most reactors are designed to reach some sort of defined environmental steady state, and water storage should be at a steady state of zero. They are particularly designed to maintain a niche that is consistent in nutrients, temperature, etc. *Note: Fed batch reactors, and other examples, are discontinuous, and the technology would not work in these settings*. For continuous reactors, this niche/environment created by engineers selectively drives the microbiome, which is functionally critical to the reactor's particular application (biofuel

production, or bioremediation, or wastewater treatment, or water storage – this last example should have almost zero nutrients, and microbial counts). The technology is fundamentally reliant on the fact that it must be optimized for the particular steady state of each application. The alarm that is described is designed so that the thresholds can be input for each application. If any of those factors that influence the microbiome change, it will immediately alert personnel. We clarified this in the Materials and Methods [Line 276-277]

We interpret the reviewers question to consider how quantifiable these steady states and disturbance thresholds are, in real-world applications. How effectively this will translate to real-world scenarios is the next step in the study, which we are dabbling in already. However, we suspect that many industrial settings – particularly those with very controlled influent such as biofuel reactors, etc. - will have enough metabolic consistency to establish a baseline and a positive and negative threshold to alert personnel of disruptions.

We believe the this supports the necessity of publishing such proof-of-concepts even more, since we are limited in capacity. We have already begun to dabble in real-world testing, but if the article is published, and the idea gains traction, there will be great value to wider groups running industrial trials and exploring the consistency and variability of CO_2 production in the many industrial applications we envision (from biofuel reactors, to wastewater treatment, to simply microbial contamination in dental chair water circulation tubing).

We cannot make a literature survey of this, since there are few papers exploring the consistency of CO_2 production in unique reactor applications, and the nuance of CO_2 disturbance necessary to indicate a reactor disruption that is concerning for the maintenance personnel of that particular application. It is too specific, too diverse and would need focused work. We would hope that publishing these proof-of-concept papers, and our own increasing traction in the field of application, will inspire wider data to create a collaborative *in situ* body of work supporting the development and targeting of this technology in industry.

This comment, and the astute comment below, inspired the addition of some lines expounding on the benefits and limitations of application [Line 542-547, 552-561, 563-570]. We hope these address the reviewers concern.

Application – What is unclear from the work is the extent to which the CO_2 monitoring system can help in application. The data show that the microbial community respond to perturbations established by adjusting chlorine concentration and pH, but what about monitoring a real plant? How much variability is present in the CO2 concentrations, and what is the time-scale of this variability compared to the time-scale of an upset in the community? Would upsets be detected earlier using this type of CO2 monitor? Also, how do the authors envision going from a beaker type test to a large, open top aeration tank?

We would like to thank the reviewer for their vision towards the use of the technology, and their rigorous considerations of application challenges. We have described some potential design adjustments for upscaling and in-field applications [Line 561-570]. We have even

begun testing some of these. However, this data would make the article very heavy and confusing, and we envision the various bodies of research as multiple follow-up studies in their own right. This proof of concept paper would support funding applications, and allow other scientists to work alongside us in testing real-world applications.

Is this a sample and test method, or is there potential for real time monitoring of the process?

The technology used in this application is a real-time CO_2 monitoring device: it passes the gas from the reactor headspace over an analyzer. For this study, we used relatively expensive Li-Cor sensors. We used 2, and interchanged them regularly, assessing the variation to evaluate sensor drift and variability between sensors [line 132-134]. However, another study in our lab has redesigned it at much lower cost, already making the tech more accessible, although the thresholds would need to be tested for these purposes. This type of continuous measurement does not need an array of sensors, just a pump passing gas over one sensor, for open loop systems measuring higher metabolic rates.

For lower metabolic rates, like the contamination events in cleaner water, we designed a closed-loop system with much lower detection limits, that measures accumulation and flushes the reactor headspace at a pre-determined frequency. In this study, we only measured the accumulation. We have since had an Honours student develop an automated flushing system, since accumulation must be measured per day (briefly mentioned in Line 410 – not published in this paper). Perpetual accumulation is not feasible. Thus, by developing this regulated flushing system, a continuous system is converted to a system that provides accumulation data per day, which should be zero for a clean water system. It is not a complicated adjustment, but, again, thresholds are application specific and must be extensively optimized. It becomes heavy to explore all of the further design and optimization elements in one paper. We envision the current manuscript as the first step, and future applications expounded in subsequent papers. By publishing this, we invite more scientists and engineers into the expansion of the work.

If the latter, is there a need for an array of sensors, or some sort of gas collection device?

As mentioned, for most bioreactors, a completely sealed headspace is not necessary for the success of this technology. In this experiment, the headspace was trapped, but open to ambient CO_2. There is enough CO_2 production to simply pass the gas over the sensor, for real-time monitoring. If monitoring drinking water storage tanks – which are used extensively in our local area after a recent drought drove our city to alternative water storage methods, the gas is trapped above the headspace is already sufficient for cumulative closed-loop studies. We also mention a small diversion of the liquid matrix to employ in the context of open-topped aerated reactors, into a sealed vessel, in parallel with the larger reactor, constantly mixing [Line 561-563]. We also envisioned tubes around dental chair water circulation mechanisms, similar to CEMS used in our lab to measure microbial metabolism in biofilms grown within tubes. [Line 564-566] We agree that a deeper discussion of the mechanistic application of the technology strengthens the paper.

I raise these questions because the manuscript implies that the method can help in industrial settings without actually testing industrial settings.

We agree that the implications from research to application were too strongly and confidently stated, within the limitations of this confined first step. We are very excited about the potential of this technology in industrial settings, and have added a paragraph describing potential applications in more detail, as extensively described above. But we also made it clear that no conclusions can be made about application without *in situ* testing of the technology [line 568-570].

River water vs activated sludge – the manuscript is often redundant in terms of the comparison between the two waters. The rationale for investigating these two end members of microbial activity are clear and can be stated once – ideally in the methods section. As I understand the manuscript, the results using the river water were not meaningful (Lines 290-298 suggest confounding factors limited the sensor or its interpretation). Unfortunately, this makes inclusion of the river water concept and data substantially less important. I recommend that the river water aspect be removed from the manuscript, or repeated using an experimental approach which resolves the issues related to the apparatus.

We did conclude that it is only useful for greater pollution events in monitoring clean water storage, but did describe the limits at which those events would be detectable by the technology, in our setting. These are certainly of interest in certain fields where water quality standards are not as stringent (industrial water re-use, agricultural water usage), it is just a limiting factor for drinking water treatment. This was mentioned in Line 441-445. It is even useful to monitor drinking water storage tanks in rural settings with this type of technology. More nuanced systems are needed to monitor to drinking water standards (ie. alerting personnel if coliforms increase from zero to 10 CFU/mL, which has already exceeded the national drinking water standards). However, those tests are usually time-consuming, demand expertise and are expensive. This technology will alert personnel of larger disturbances quickly.

This technology was developed for an EU project, which involved implementing a decentralized water treatment system, based on ozone treatment, serving villages of 300 people in remote areas in Africa. The water, post-treatment, passes through storage tanks before distribution. Monitoring the CFU in the tanks via genetics or culturing is the only way to detect fluctuations above drinking water quality levels, resolving the difference between 0 and 10 CFU/mL. However, those monitoring techniques are difficult to access in remote areas, and in poverty. This technology, with a low demand for expertise and electricity, would be ideal to let staff know immediately if something in the drinking water treatment system fails on a larger, coarser scale. Based on this, we would appreciate leaving the water results in, as it may assist future researchers developing the tech further for this application. This application was our most passionate vision, and we would be sad to remove it. Although it might not have application in first-world drinking water monitoring, which is already high tech and relies on extremely resolved data, we are developing tech for our context, which needs

to be much more flexible and robust. We are promoting the notion of mixed methods, maintaining high standards of rigor, but supplementing it with robust, coarser systems where that type of rigor is unaffordable and inaccessible. Or at least, supplementing it in scenarios where more expensive tech cannot be afforded at the frequency of municipal labs, for instance. This has been expounded further in Line 446-454.

We also believe that negative results are as informative as positive results, as it prevents redundancy in future testing.

Thus, we would appreciate retaining this section, but would be willing to remove it if the reviewer still has qualms. We agree that, in the context of a first-world drinking water treatment plant, this would be fully redundant, as the reviewer pointed out. We hope our additions have clarified this.

Minor items

Figure 3a – the use of predicted here for the modeled CO2 is incorrect. The manuscript states that the modeling results shown in figure 3a are a fit. Predictions have no fitted parameters (i.e., parameters are determined independent of the experiment being modeled). The modeling results for the 0.25 L case should be smooth though there appears to be a small increase then decrease in modeled CO2 around 2 hr. If this feature is real (i.e., the plot is small and the data line may be misleading the eye), then what in the model can account for the presence of this type of behavior? See above concern about the two parameter fit.

Thank you, the term “predicted” has been replaced with “model fit”.

The small perturbations in the model are due to variations in the atmospheric CO_2 levels, which was incorporated into our mathematical model as described in the supplementary material. In our experimental setup, atmospheric air flowed into the sample headspace as headspace gas was withdrawn for CEMS analysis. The CO_2 levels in the headspace would therefore be affected by the atmospheric CO_2 concentration.

A separate CEMS device was used to monitor atmospheric CO_2 concentrations while the headspace experiments were conducted. The measured time varying atmospheric CO_2 concentration was used as input to the dynamic model, represented as $p_{CO2,atm}$ in eq. A3 of the supplementary materials S1. An increase in atmospheric CO_2 would therefore lead to an increase in the $p_{CO2,atm}$ value used as input to the model, which subsequently led to an increase in the modelled headspace CO_2 concentration.

Reviewer #3: This manuscript provides a methodology to detect aberrations in microbial growth in an industrial setting. The advantage of CEMS is its utilization of sustainable, existing technology to function as a first-pass ‘canary’. Overall

the manuscript is technically sound after the first revision. However, we would like to see more details on the points that we made below.

1. For figure 3a & b:

For Figure 3a, there is a discontinuation of the undiluted 0.90 L headspace experimental data between 3 - 6 hr point. We would like to see more explanation on how the measurement was done in specific.

The discontinuation was simply due to a power outage. This is a regular occurrence that we need to navigate, due to national rolling blackouts.

(https://www.bloomberg.com/news/articles/2020-07-12/south-africa-s-eskom-extends-blackouts-due-to-supply-constraints).

We have included this in the Materials and Methods and in the Figure description (Line 139-141, Line 344). Since out primary vision is to employ this technology in resource limited areas, the aim is to run it off solar power, which is an abundant resource in our context. This was already incorporated in the text in Line 91.

2. For the description of figure 3 in the main manuscript, refer to the mathematical appendix for the equations and parameters to more clearly explain the regression.

Appropriate references to the supplementary materials were provided.

3. L 416-421: The authors stated that this work demonstrates an early warning system for such significant contamination events (20 CFU/ml to 10300 CFU/ml). It would make a stronger argument if the authors explain what other methods are there for this kind of detection and give a comparison why this system would be better than the others.

This is a very good point. The strongest alternatives for rapid detection that really meet the requirements for resolution at drinking water standards with speeds lower than the days required for typical culturing are qPCR and enzyme assays. We already discussed some of this competing technology and compared thresholds and benefits in Line 86-88, Line 426-432 and Line 583-592. We hope the sentences in Line 432-439, 446-454 and 572-592 describe our vision for a mixed methods approach to water monitoring. We suggest that it doesn't compete with techniques of greater data resolution, but supplements precision techniques with the benefits of real-time, online data access with alarms. However, we agree that more depth is needed in understanding competing options, and added this as described in the sentences above, hopefully to a satisfactory depth. We also adjusting the references. Both reviewers pointed this out: we thank you for underlining the need for greater depth in this discussion.
