# Supplementary material for: Canary in the coliform mine: Exploring the industrial application limits of a microbial respiration alarm system
Source: PLoS One. 2021 Mar 4;16(3):e0247910. doi: 10.1371/journal.pone.0247910 (PMC7932117; doi:10.1371/journal.pone.0247910)
Supplement: Attachment — Submitted filename: Response to Reviewers.docx [file pone.0247910.s002.docx]

Journal Requirements:

When submitting your revision, we need you to address these additional requirements.

1. Please ensure that your manuscript meets PLOS ONE's style requirements, including those for file naming. The PLOS ONE style templates can be found at

<https://journals.plos.org/plosone/s/file?id=wjVg/PLOSOne_formatting_sample_main_body.pdf>

and

<https://journals.plos.org/plosone/s/file?id=ba62/PLOSOne_formatting_sample_title_authors_affiliations.pdf>

Thank you for the guidance. We have gone carefully through formatting, and have met the stipulated requirements. If further editing is necessary, we are happy to do so.

As primary and corresponding author, I made the mistake of not using track changes as I edited.

However, the only edits we made were those recommended by the reviewers and described below (including Line numbers of edits). In addition, I formatted according to the links provided (headings, figure and table captions, and references). In addition, all figures were corrected using PACE.

I highlighted the changes in yellow, to account for the mistake of not using track changes before saving the new, edited document.

Additional Editor Comments (if provided):

This manuscript describes an early-warning system for microbial disturbances in industrial settings by exploring microbial biofilm growth and metabolic responses. It is an interesting paper. However, the experiment design and statistical analysis need to be improved. I would like to receive the revised paper if you can address the reviewer's comments.

We thank the editor for the opportunity to respond to the reviewer’s comments and resubmit. We hope we have addressed all comments to your satisfaction, and are available for follow up if further issues need clarification.

The reviewer’s comments were astute, and we are grateful for the improvement of the manuscript through the editorial process.

L122-125	It is stated that river water was used to assess “very low levels of microbial contamination”. However, the river is from a highly polluted source as stated earlier in the manuscript and as is supported by the chemical data that show very high COD. It needs to be explained here that the river water will be added in very low concentrations to pure water in order to mimic clean water.

We thank the reviewer for this clarification, and have adjusted the sentence as follows:

“This polluted river water was diluted with pure water to mimic a range of pollution levels, from low to high.” (Line 118-119)

L131-141 , 149-161	Its not stated explicitly how many sensors were used so I assume it was just one, and the sensor was used in sequential experiments to measure differences. Standard deviations were calculated from the STD of measurements obtained as a time series during a single sensor run.

The problem with this is that any temporal drift in the sensor output will be interpreted as a difference between treatments. It also violates the assumptions of a t-test which requires independent measurements when in this case, all data for the same treatment were autocorrelated. It is presumably this that accounts for the supposed significant differences between some treatments in Fig 2 despite near identical means. Instead, the figure should show the average and STD derived from repeated (independent) experiments with the treatments run in randomized order to counteract systematic biases caused by drift. If the experiment wasn’t repeated then variance cant be assessed.

Two sensors were used, rather than one, but the reviewer is correct in this statistical observation. The sensors were regularly interchanged throughout all the runs, between control and experimental reactors, and between high and low CO_2_ accumulation, in order to assess drift. We can confirm that sensor drift is negligible. Both this random, regular exchange between sensors during experimental work confirms this, and, as stated earlier in the manuscript “Respiration was monitored in reactor headspaces, which were open to the atmosphere via a defined inlet port. Baselines were predictable at 385 ppm CO2 ± 45 ppm for the open-loop system, with variation attributed to human activity in the vicinity increasing CO2 levels during the day. Closed-loop baselines had a similar standard deviation if measured at separate instances over different days, but a variation of less than 5 ppm over days if sealed.” The low variance under similar controlled conditions on different days allows us to safely conclude that any sensor drift is negligible. We thank the viewer for this meticulous caution, and have added a sentence about using two sensors with random interchange during experimentation (Line 132-137), for replicability of the work.

We agree that employing a t-test was inappropriate on our part, due to the use of correlated data. Since the assumptions of a t-test were violated, we have removed the significance indicators on the graph and have removed that statistical description in ‘Material and Methods’. However, based on the established low variance in sensor measurements and the noticeable difference between the measured respiration for the Undiluted River Water sample in the 0.9 L headspace experiment and all five other experiments, we can still confidently conclude that microbial activity is detectable and a larger headspace volume enhances measurement sensitivity, thus supporting the methodology developed subsequently.

L380	“Headspace CO2 concentration had a linear correlation with the 380 dilution of the river water,” between 25 and 100% strength (Fig 4; R2 = 0.986) and all other data with R2 in Fig 4.
AND
L287	“This technique confirmed the metabolic profile with tight correlation to CO2 data (R2=0.99; Fig 4).

The R2 values reported are much higher than would be expected from the scatter seen in the data in the figures. The only explanation I can think of is that the data were averaged before calculating R2, which is the difference between the two plots shown here: [https://i.imgur.com/GDZmrjv.png](https://eur03.safelinks.protection.outlook.com/?url=https%3A%2F%2Fi.imgur.com%2FGDZmrjv.png&data=04%7C01%7C%7C6e662d4c15fe46002d3a08d886e0848b%7Ca6fa3b030a3c42588433a120dffcd348%7C0%7C0%7C637407646148647990%7CUnknown%7CTWFpbGZsb3d8eyJWIjoiMC4wLjAwMDAiLCJQIjoiV2luMzIiLCJBTiI6Ik1haWwiLCJXVCI6Mn0%3D%7C1000&sdata=P3N3%2Be61KRF4Rz74QEEFfLoCocufCG7WuZalFkmNt38%3D&reserved=0) . This artificially deflates variance by performing stats on already processed data.

The reviewer is correct, and the statistical values were calculated based on averages. In an attempt to fit all of the graphs into an aesthetically streamlined format, presenting multiple assessments per quadrant, the statistical analyses were incorrectly collapsed into a format that, as the reviewer points out, deflates variance.

We have expanded them all as recommended by the reviewer and avoided passing the correlations through zero. We kept them on separate graphs for ease of audience interpretation, as single images become too busy. (Lines 350-363). We apologize for the conflation of the statistical values, and are grateful to the reviewer for encouraging the more rigorous analysis and stringent interpretation.

L414	Fig 5 caption doesn’t match the figure. I don t understand what Fig 5 is showing.

Thank you for bringing the lack of articulation to our attention. We’ve expanded the figure caption to provide a clearer description of the figure. We hope this brings clarity: if it is still unclear, we are happy to edit further. (Line 396-401).
